# Supplementary material for: Genome mining yields putative disease-associated ROMK variants with distinct defects
Source: PLoS Genet. 2023 Nov 13;19(11):e1011051. doi: 10.1371/journal.pgen.1011051 (PMC10695394; doi:10.1371/journal.pgen.1011051)
Supplement: S1 Fig — The growth of yeast containing a vector control, wild-type ROMK, or the indicated mutation in the context of an activating mutation, K80M, was measured in liquid medium containing 10mM KCl. Note that the growth phenotype of a mutant should be compared to the “ROMK-K80M” curve at the top. OD600 readings were recorded every 30 min for 23.5 hrs. Data represent the means of 8 replicates, ± S.E (error bars). A summary of these data is shown in S3 Table. The top graph contains the growth curves for all variants, but since many overlap at OD ~0.1 (red scale bar), this section of the graph was magnified for clarity (see graph at the bottom). (DOCX) [file pgen.1011051.s001.docx]

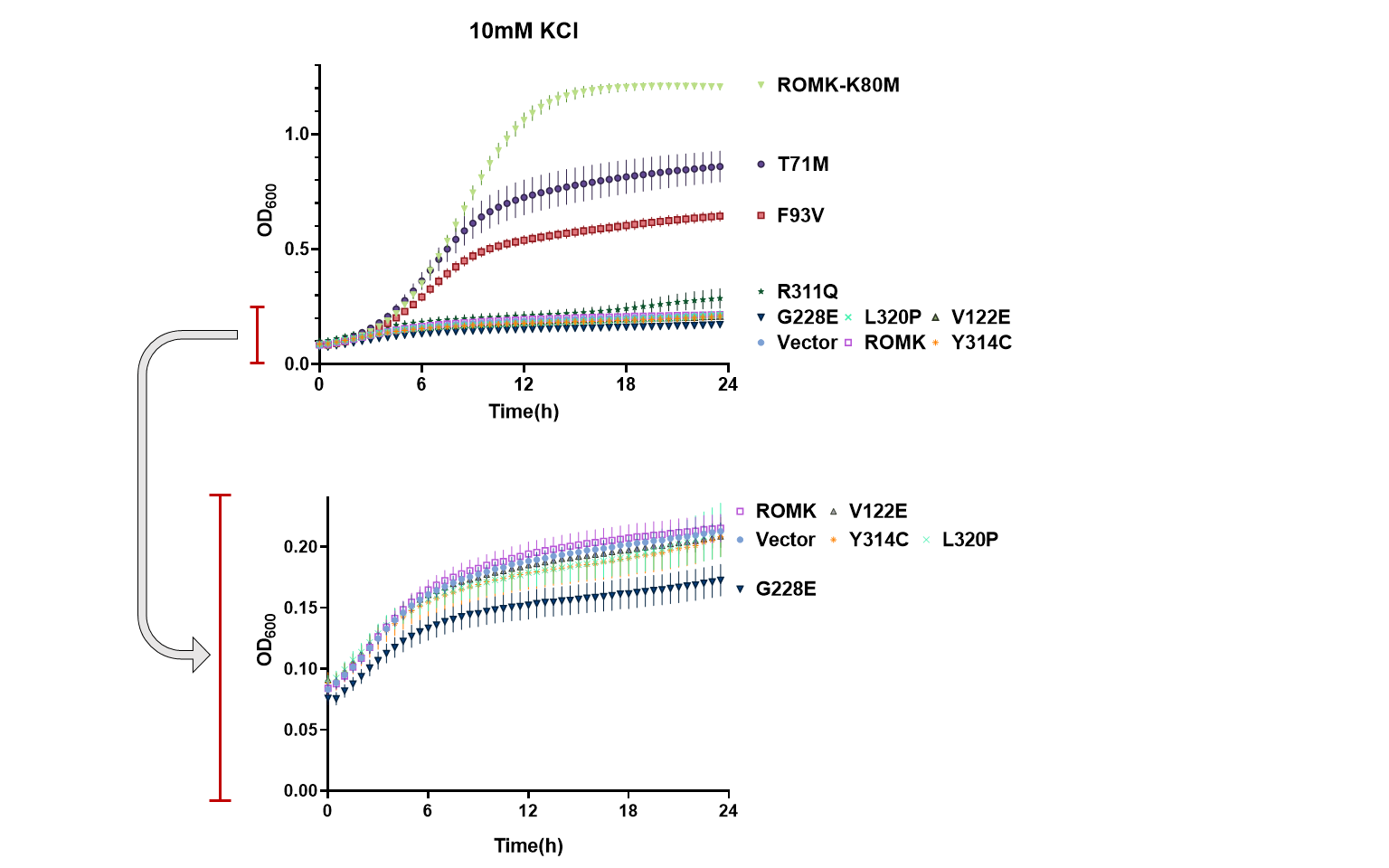


## **S1 Fig. Growth assays of yeast expressing select TOPMed mutations in the context of the K80M allele.**

The growth of yeast containing a vector control, wild-type ROMK, or the indicated mutation in the context of an activating mutation, K80M, was measured in liquid medium containing 10mM KCl. Note that the growth phenotype of a mutant should be compared to the “ROMK-K80M” curve at the top. OD_600_ readings were recorded every 30 min for 23.5 hrs. Data represent the means of 8 replicates, ± S.E (error bars). A summary of these data is shown in **S3 Table**. The top graph contains the growth curves for all variants, but since many overlap at OD ~0.1 (red scale bar), this section of the graph was magnified for clarity (see graph at the bottom).
