# Supplementary material for: Genome mining yields putative disease-associated ROMK variants with distinct defects
Source: PLoS Genet. 2023 Nov 13;19(11):e1011051. doi: 10.1371/journal.pgen.1011051 (PMC10695394; doi:10.1371/journal.pgen.1011051)
Supplement: S2 Fig — REVEAL: Biobank is a platform built upon SciDB, a computational database ideal for large scale linear algebra operations, and is comprised of an R-programmed API and Graphic User Interfaces (GUIs) for cohort selection and PheWas visualization. This platform has multiple features: elastic scaling (Burst Mode) for efficient and cost-effective analyses; Bridge, a cloud-optimized array format; and flexFS, a networked POSIX compliant filesystem for working with big data in the UK Biobank. See Materials and Methods for details. (DOCX) [file pgen.1011051.s002.docx]

**
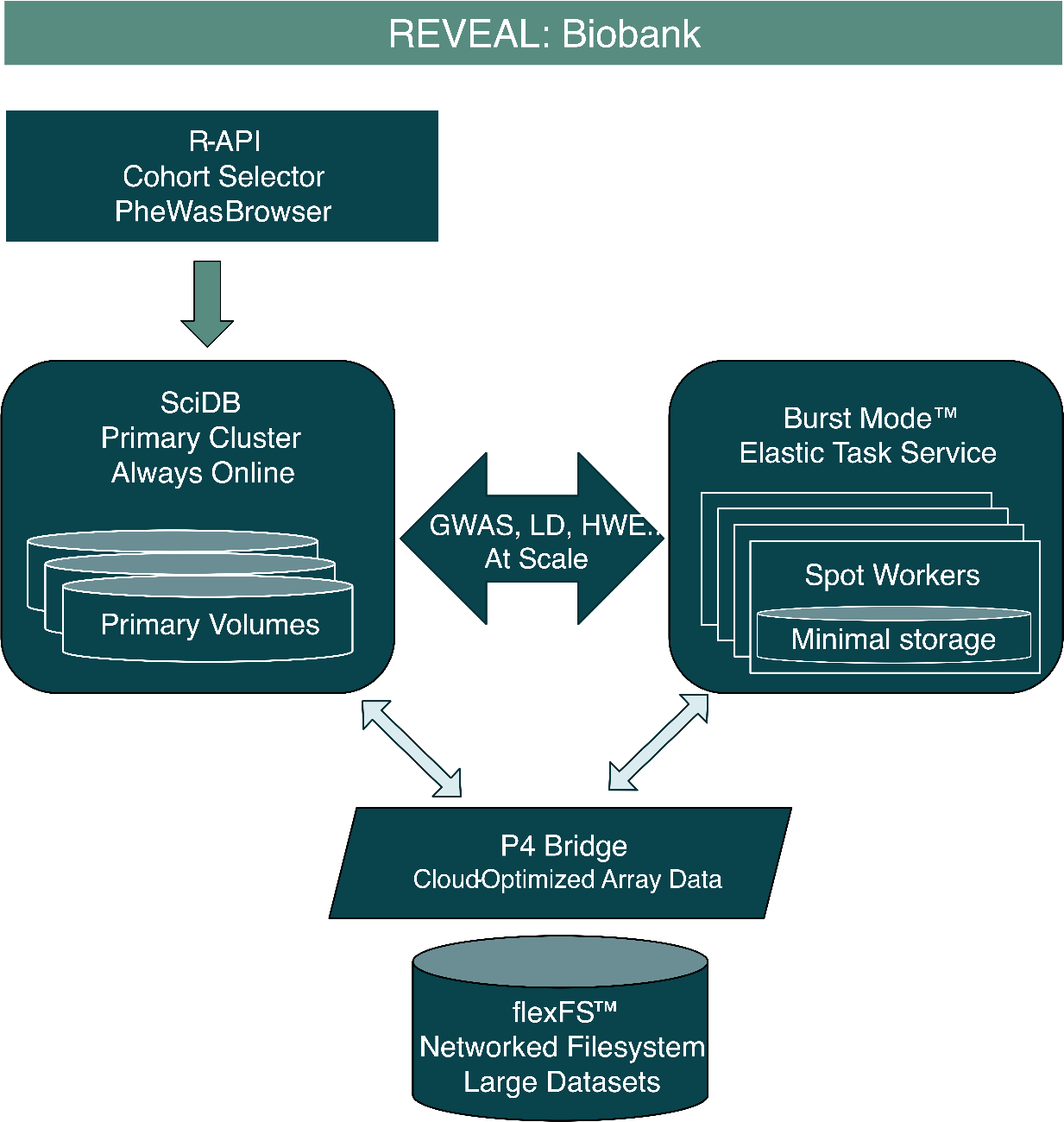
**

## **S2 Fig. REVEAL: Biobank platform.**

REVEAL: Biobank is a platform built upon SciDB, a computational database ideal for large scale linear algebra operations, and is comprised of an R-programmed API and Graphic user interfaces (GUIs) for cohort selection and PheWas visualization. This platform has multiple features: elastic scaling (Burst Mode) for efficient and cost-effective analyses; Bridge, a cloud-optimized array format; and flexFS, a networked POSIX compliant filesystem for working with big data in the UK Biobank. See **Materials and Methods** for details.
