# Supplementary material for: Genome mining yields putative disease-associated ROMK variants with distinct defects
Source: PLoS Genet. 2023 Nov 13;19(11):e1011051. doi: 10.1371/journal.pgen.1011051 (PMC10695394; doi:10.1371/journal.pgen.1011051)
Supplement: S3 Fig — Stability assays performed in yeast expressing the wildtype ROMK, or ROMK carrying the mutation G228E, L320P, or N377K. To assess the effect of the yeast AAA+-ATPase Cdc48 on protein degradation, a temperature sensitive yeast strain (cdc48-2) was used. Yeast cultures were grown to mid-log phase (OD600 0.7–1.5) at a permissive temperature, diluted, and incubated at a non-permissive temperature of 39°C for 2 hours before adding cycloheximide. Cells were then processed, and immunoblot analysis was performed (see Materials and Methods). Representative immunoblots are shown, and graphs show the percentage of the protein remaining over time, compared to the 0 min (m) time point, as quantified by ImageJ (ver. 1.53c). Graphs were made using GraphPad Prism (ver. 9.5.0), and data represent the means of at least three independent experiments, ± S.E. (error bars). For each experiment, a representative immunoblot is shown. (DOCX) [file pgen.1011051.s003.docx]

**
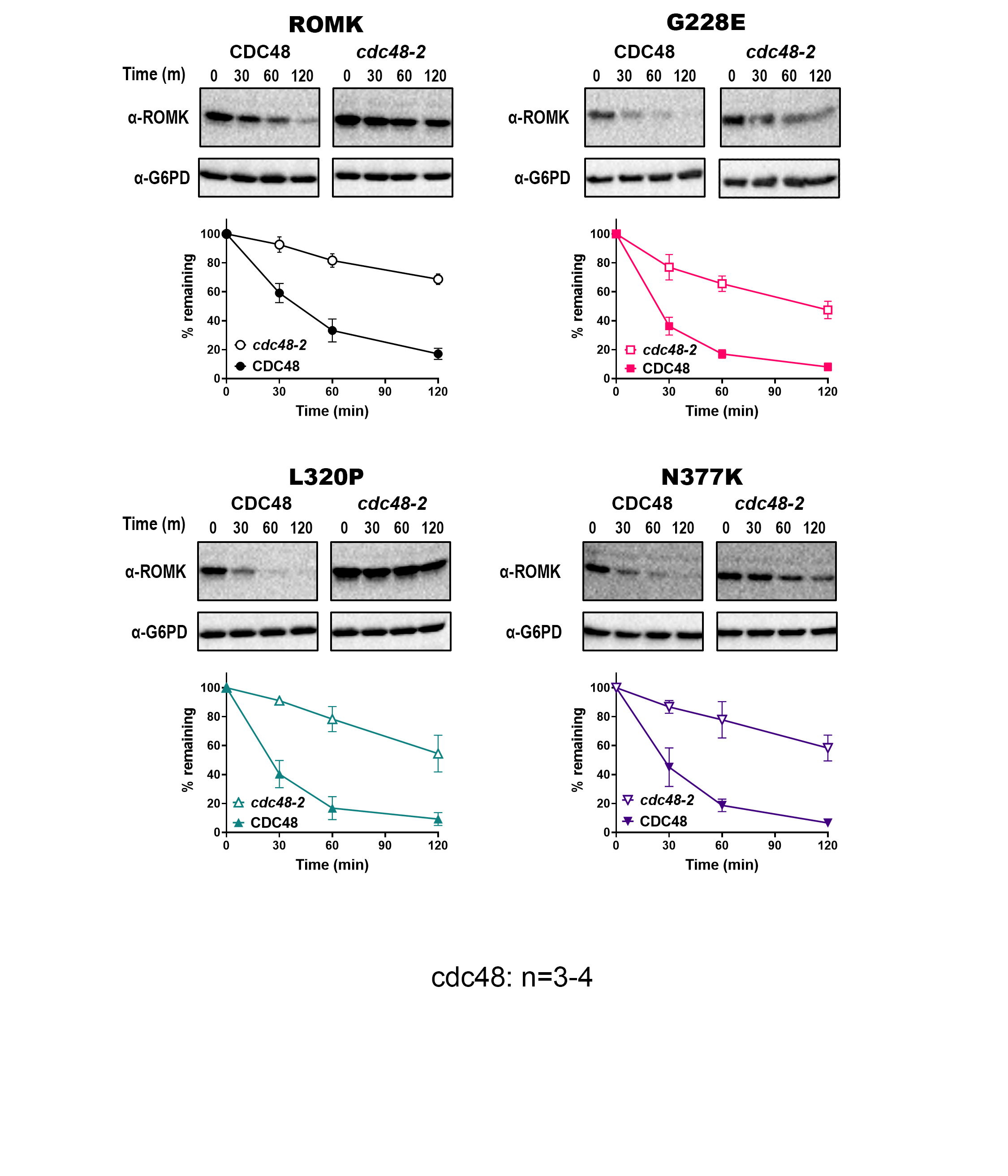
**

## **S3 Fig. The degradation of select ROMK mutants is Cdc48-dependent in yeast.**

Stability assays performed in yeast expressing the wildtype ROMK, or ROMK carrying the mutation G228E, L320P, or N377K. To assess the effect of the yeast AAA^+^-ATPase Cdc48 on protein degradation, a temperature sensitive yeast strain (*cdc48-2*) was used. Yeast cultures were grown to mid-log phase (OD_600_ 0.7-1.5) at a permissive temperature, diluted, and incubated at a non-permissive temperature of 39°C for 2 hours before adding cycloheximide. Cells were then processed, and immunoblot analysis was performed (see **Materials and Methods**). Representative immunoblots are shown, and graphs show the percentage of the protein remaining over time, compared to the 0 min (m) time point, as quantified by ImageJ (ver. 1.53c). Graphs were made using GraphPad Prism (ver. 9.5.0), and data represent the means of at least three independent experiments, ± S.E. (error bars). For each experiment, a representative immunoblot is shown.
