# Supplementary material for: Genome mining yields putative disease-associated ROMK variants with distinct defects
Source: PLoS Genet. 2023 Nov 13;19(11):e1011051. doi: 10.1371/journal.pgen.1011051 (PMC10695394; doi:10.1371/journal.pgen.1011051)
Supplement: S4 Fig — Representative cell-surface biotinylation assay showing the surface expression levels of the indicated ROMK variants expressed in HEK293 cells. The experimental set-up is identical to the experiment shown in Fig 7, except data for N377K are included. (DOCX) [file pgen.1011051.s004.docx]

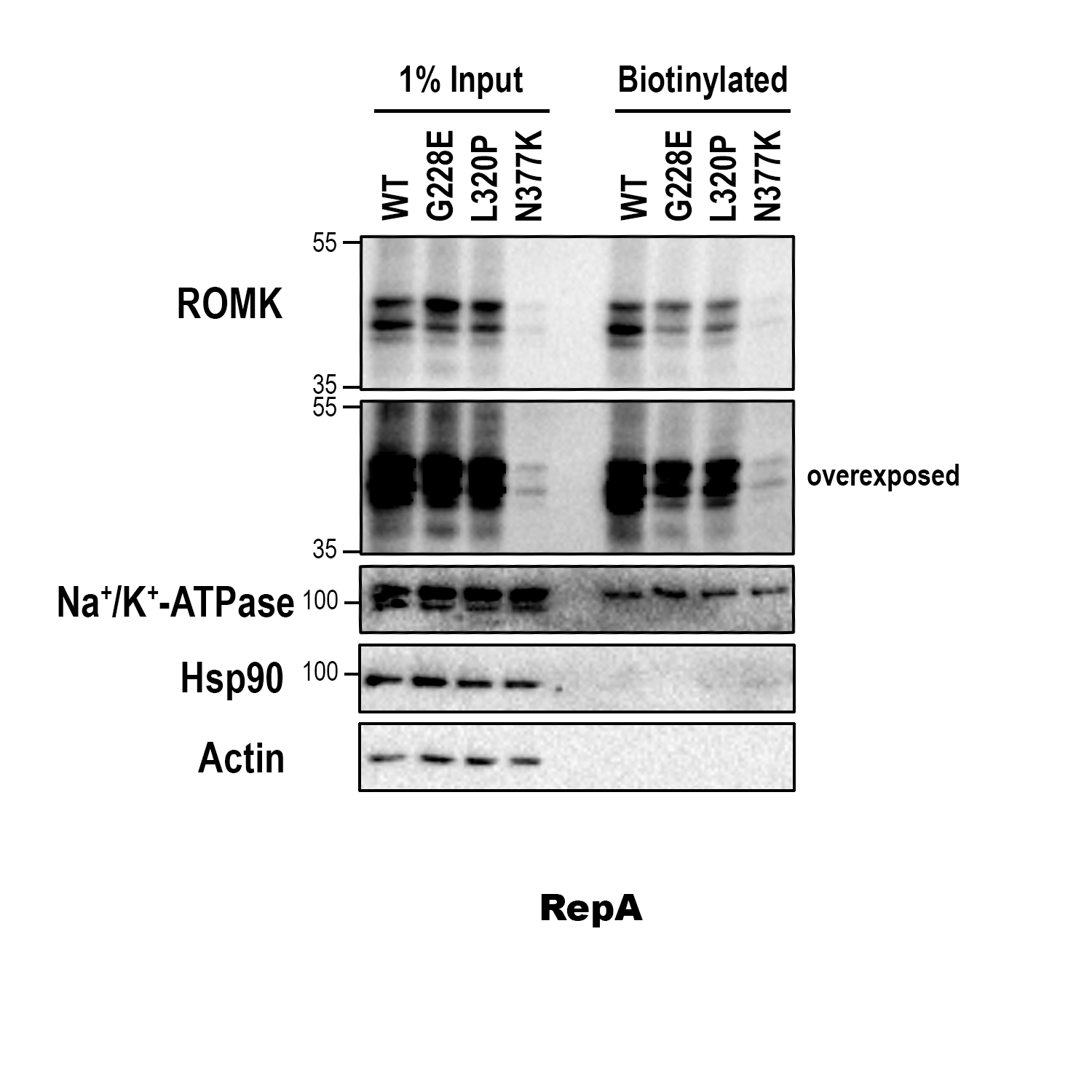


## **S4 Fig. Select mutants reduce ROMK protein levels at the cell surface.**

Representative cell-surface biotinylation assay showing the surface expression levels of the indicated ROMK variants expressed in HEK293 cells. The experimental set-up is identical to the experiment shown in **Fig 7**, except data for N377K are included.
