# Supplementary material for: Genome mining yields putative disease-associated ROMK variants with distinct defects
Source: PLoS Genet. 2023 Nov 13;19(11):e1011051. doi: 10.1371/journal.pgen.1011051 (PMC10695394; doi:10.1371/journal.pgen.1011051)
Supplement: S5 Fig — Homology model shows the pore of the tetrameric ROMK channel, as viewed from the cytoplasmic side. Potassium ions (spheres in the center of the pore) are shown in salmon and outlined with dotted black lines. Spheres depicting the positions of T300 (A), T300I (B), and T300R (C) are in green, cyan, and magenta, respectively. Only residues 184–364 of each chain are shown for clarity. The homology model was built based on the crystal structure of Kir2.2 (PDB ID: 3SPG), which is 47.42% identical to ROMK1. Images were rendered using PyMOL (ver. 2.6.0). (DOCX) [file pgen.1011051.s005.docx]

**
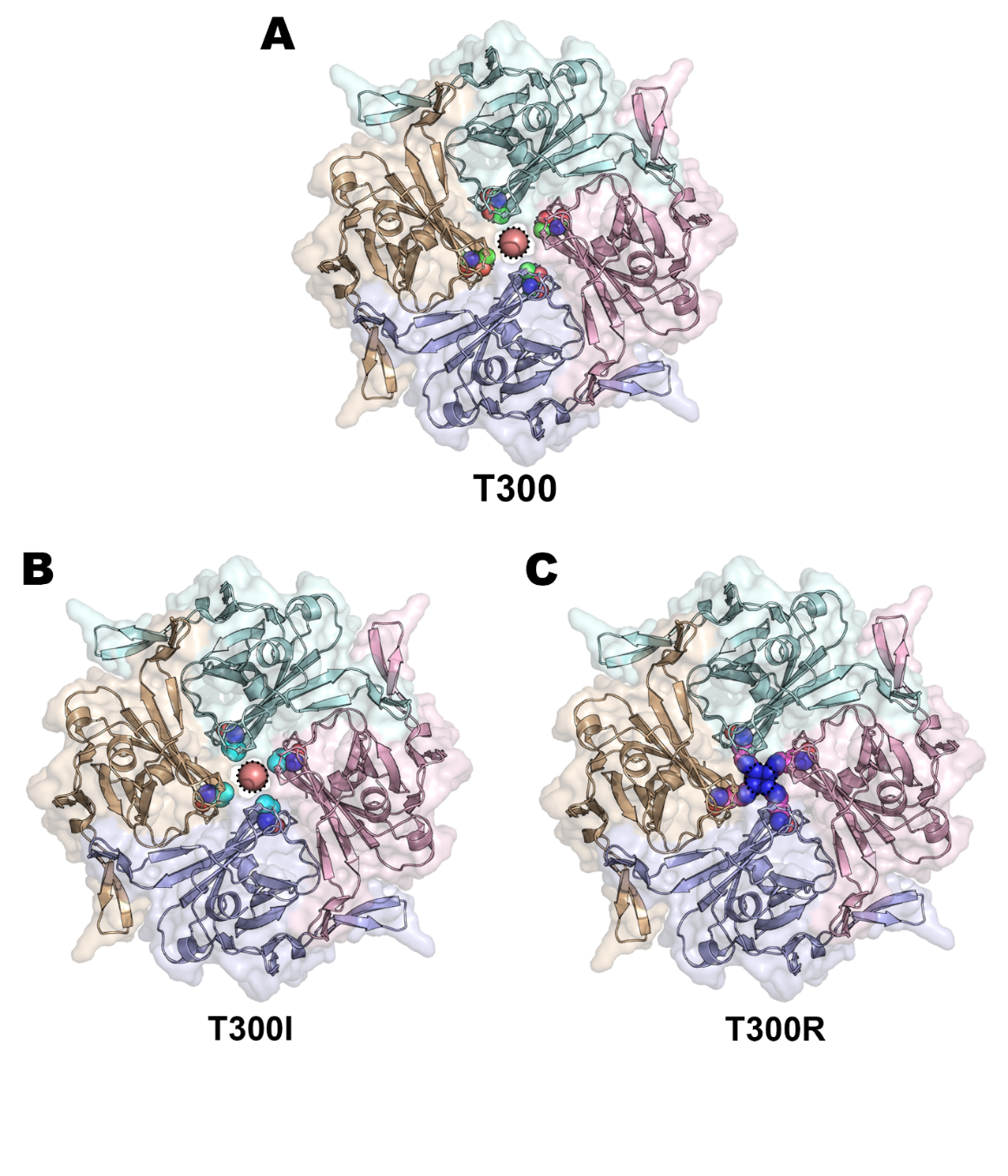
**

## **S5 Fig. Structural modeling suggests the T300R mutation occludes the cytoplasmic pore.**

Homology model shows the pore of the tetrameric ROMK channel, as viewed from the cytoplasmic side. Potassium ions (spheres in the center of the pore) are shown in salmon and outlined with dotted black lines. Spheres depicting the positions of T300 **(A)**, T300I **(B)**, and T300R **(C)** are in green, cyan, and magenta, respectively. Only residues 184-364 of each chain are shown for clarity. The homology model was built based on the crystal structure of Kir2.2 (PDB ID: 3SPG), which is 47.42% identical to ROMK1. Images were rendered using PyMOL (ver. 2.6.0).
