# Supplementary material for: Genome mining yields putative disease-associated ROMK variants with distinct defects
Source: PLoS Genet. 2023 Nov 13;19(11):e1011051. doi: 10.1371/journal.pgen.1011051 (PMC10695394; doi:10.1371/journal.pgen.1011051)
Supplement: S6 Fig — ROC curves (in blue solid lines) were computed for the 17 selected mutations from TOPMed and ClinVar, along with a previously published Y314C mutation [15]. True positive and false positive designations were determined based on yeast growth data from Fig 2 and S3 Table. Mutations were considered deleterious if they resulted in a relative endpoint of OD600 <0.9. Otherwise they were considered neutral. The area under the ROC curve (AUROC or AUC) were then calculated using the composite trapezoidal rule. Five ROC curves with their corresponding AUROC were computed: (A) Rhapsody using yeast growth data from Fig 2; (B) Rhapsody, with both the original yeast growth dataset and the new data using the ROMK-K80M construct incorporated. In particular, the newly observed growth defects changed the designations of two mutations (F93V and V122E) to deleterious; (C) Polyphen-2; (D) EVmutation; (E) EVE. For reference, a line of no-discrimination (dotted black line) is shown, which corresponds to a purely random classifier. (DOCX) [file pgen.1011051.s006.docx]

**
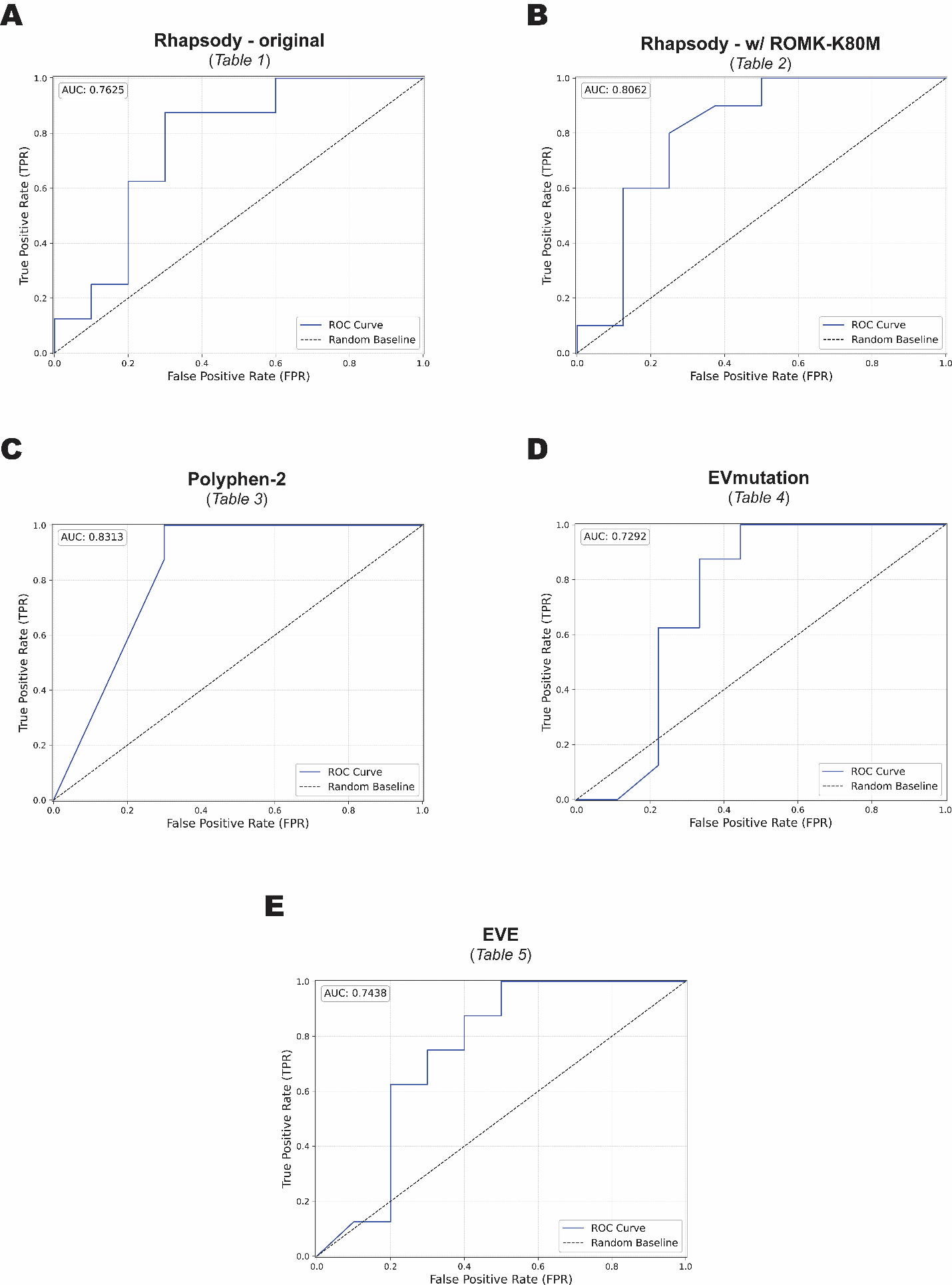
**

## **S6 Fig. ROC curves of TOPMed and ClinVar mutations in relation to their pathogenicity scores and yeast growth phenotypes.**

ROC curves (in blue solid lines) were computed for the 17 selected mutations from TOPMed and ClinVar, along with a previously published Y314C mutation (1). True positive and false positive designations were determined based on yeast growth data from **Fig 2** and **S3 Table**. Specifically, a “true positive” was assigned to the ROMK mutants with severe and moderate defects, i.e., mutations that resulted in a relative endpoint OD_600_ of < 0.9. In contrast, mutants with endpoint OD_600_ of ≥ 0.9 were “false-positive”. The area under the ROC curve (AUROC or AUC) were then calculated using the composite trapezoidal rule. Five ROC curves with their corresponding AUROC were computed: **(A)** Rhapsody using yeast growth data from **Fig 2**; **(B)** Rhapsody, with both the original yeast growth dataset and the new data using the ROMK-K80M construct incorporated. In particular, the newly observed growth defects changed the designations of two mutations (F93V and V122E) to true-positive; **(C)** Polyphen-2; **(D)** EVmutation; **(E)** EVE. For reference, a line of no-discrimination (dotted black line) is shown, which corresponds to a purely random classifier.

1. O'Donnell BM, Mackie TD, Subramanya AR, Brodsky JL. Endoplasmic reticulum-associated degradation of the renal potassium channel, ROMK, leads to type II Bartter syndrome. J Biol Chem. 2017;292(31):12813-27.
