# Supplementary material for: Genome mining yields putative disease-associated ROMK variants with distinct defects
Source: PLoS Genet. 2023 Nov 13;19(11):e1011051. doi: 10.1371/journal.pgen.1011051 (PMC10695394; doi:10.1371/journal.pgen.1011051)
Supplement: S7 Fig — Large and small colonies of trk1Δtrk2Δ yeast carrying an empty vector, the ROMK protein, or the N377K mutant were propagated on synthetic complete medium lacking leucine and containing 3% glycerol. Plates show two independent replicates, and images were taken with the Bio-Rad ChemiDoc XRS+ imager after a 7-day incubation (4 days at 30°C, 3 days at 22°C). (DOCX) [file pgen.1011051.s007.docx]

**
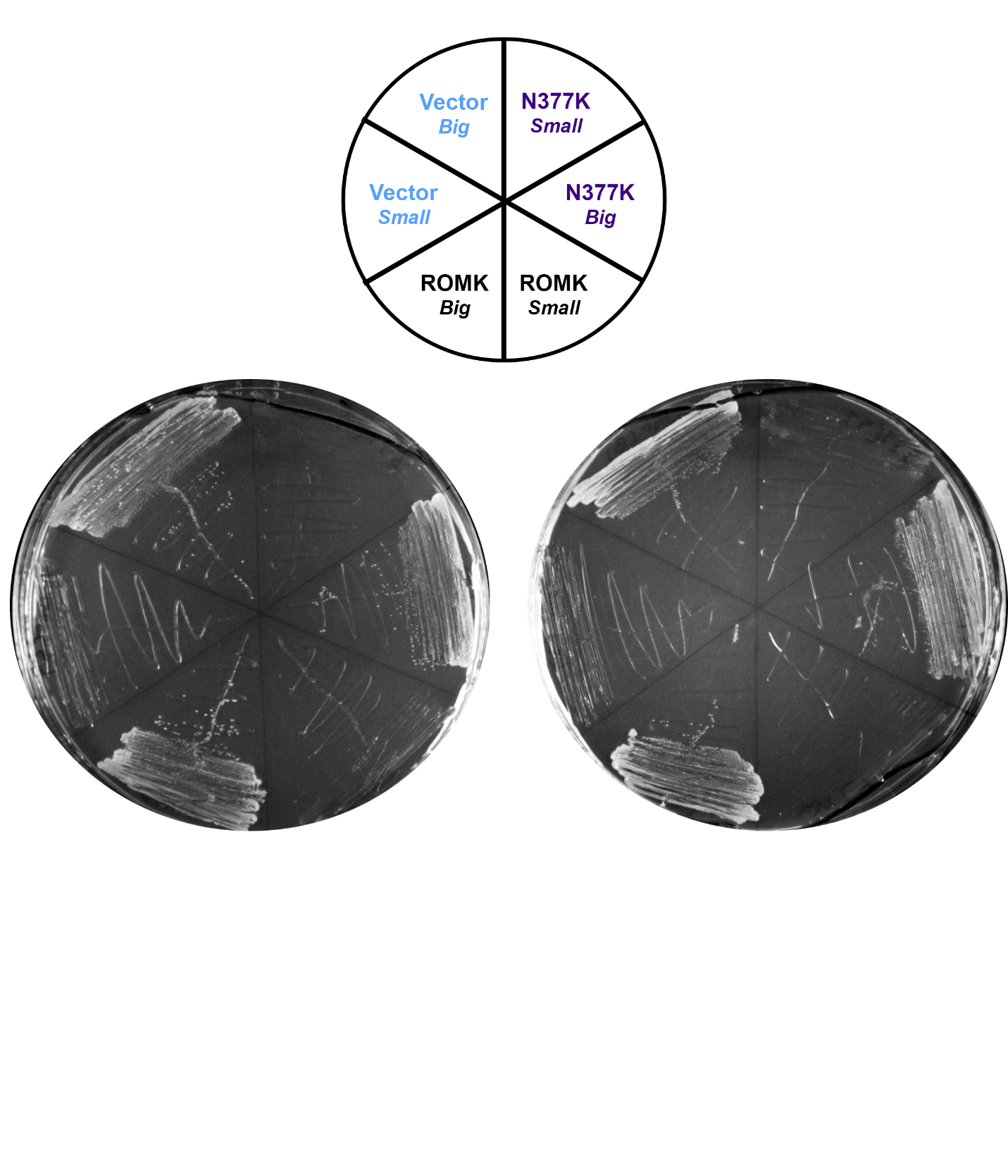
**

## **S7 Fig. Slow growth *trk1*Δ*trk2*Δ yeast colonies fail to propagate on a nonfermentable carbon source.**

Large and small colonies of *trk1*Δ*trk2*Δ yeast carrying an empty vector, the ROMK protein, or the N377K mutant were propagated on synthetic complete medium lacking leucine and containing 3% glycerol. Plates show two independent replicates, and images were taken with the Bio-Rad ChemiDoc XRS+ imager after a 7-day incubation (4 days at 30°C, 3 days at 22°C).
