# Supplementary material for: Genome mining yields putative disease-associated ROMK variants with distinct defects
Source: PLoS Genet. 2023 Nov 13;19(11):e1011051. doi: 10.1371/journal.pgen.1011051 (PMC10695394; doi:10.1371/journal.pgen.1011051)
Supplement: S8 Fig — Currents recorded by two-electrode voltage clamps (TEVC) in X. laevis oocytes as described in Fig 8 and Materials and Methods. To recapitulate heterozygosity, oocytes were co-injected with 0.5 ng each of wild-type (WT) ROMK and the indicated mutant. (A) Graph shows the Ba2+-sensitive/ROMK current in oocytes injected with the indicated cRNAs, as recorded by TEVC. (B) Normalized currents, which are defined as Ba2+-sensitive currents divided by the means of the wild-type currents. Error bars in the graphs show the means of all replicates (in parentheses), ±S.D. p-values (shown above the data) were computed using Kruskal-Wallis and Dunn’s multiple comparisons tests. (DOCX) [file pgen.1011051.s008.docx]

**
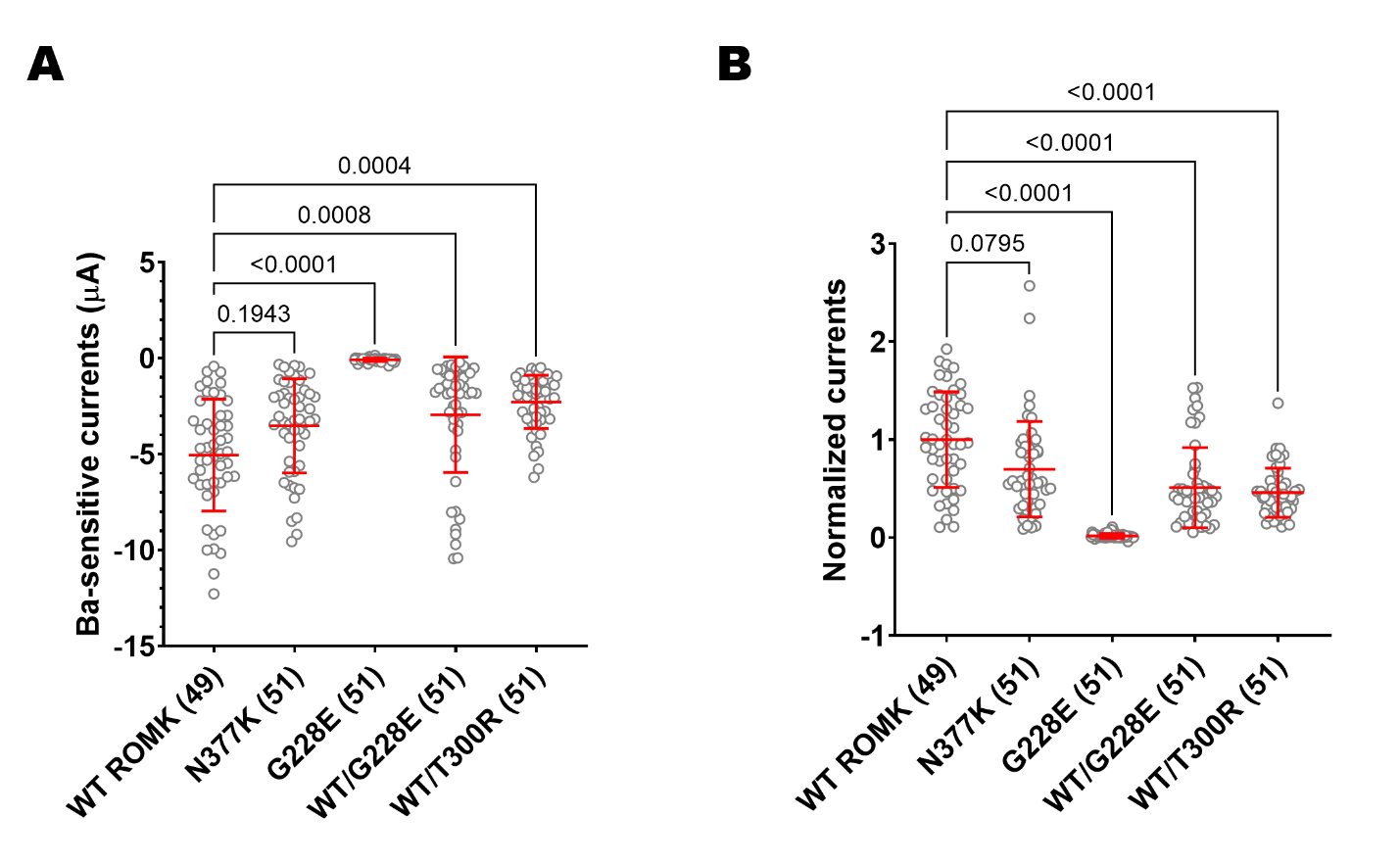
**

## **S8 Fig. Heterozygous ROMK mutants exhibit an intermediate phenotype in whole-cell currents in *X. laevis* oocytes.**

Currents recorded by two-electrode voltage clamps (TEVC) in *X. laevis* oocytes as described in **Fig 8** and **Materials and Methods**. To recapitulate heterozygosity, oocytes were co-injected with 0.5 ng each of wild-type (WT) ROMK and the indicated mutant**.** (A) Graph shows the Ba^2+^-sensitive/ROMK current in oocytes injected with the indicated cRNAs, as recorded by TEVC. (B) Normalized currents, which are defined as Ba^2+^-sensitive currents divided by the means of the wild-type currents. Error bars in the graphs show the means of all replicates (in parentheses), ±S.D. p-values (shown above the data) were computed using Kruskal-Wallis and Dunn's multiple comparisons tests.
