# Supplementary material for: Genome mining yields putative disease-associated ROMK variants with distinct defects
Source: PLoS Genet. 2023 Nov 13;19(11):e1011051. doi: 10.1371/journal.pgen.1011051 (PMC10695394; doi:10.1371/journal.pgen.1011051)
Supplement: S1 Table — Table shows the Rhapsody scores and predictions of 124 ROMK missense mutations from the TOPMed program that were analyzed in this study. The analysis was performed using a ROMK homology model that contains amino acids 38–364 (see Fig 1), so any residue outside of this range lacks a Rhapsody score and is designated “-“. “Del” indicates a substitution is predicted to be deleterious, whereas “Neu” (neutral) is predicted to have no effects on channel function. A designation of “Prob. Del” or “Prob. Neu” indicates that the Rhapsody score is close to the 0.5 cutoff for being deleterious. For example, the Rhapsody scores of I85N and F94S are 0.512 and 0.470, and thus, these mutations are categorized as “Prob. Del” and “Prob. Neu”, respectively. The mutations listed in bold were selected for growth analysis in yeast. (DOCX) [file pgen.1011051.s009.docx]

| **Mutation** | **Rhapsody score** | **Rhapsody prediction** |
| --- | --- | --- |
| A3V | - | - |
| A6W | - | - |
| R6Q | - | - |
| T11M | - | - |
| T11A | - | - |
| T17A | - | - |
| S19N | - | - |
| R25W | - | - |
| R25Q | - | - |
| K26N | - | - |
| W27S | - | - |
| V29I | - | - |
| R31C | - | - |
| R31H | - | - |
| H35N | - | - |
| R37W | - | - |
| R37Q | - | - |
| D46N | 0.694 | Del |
| G47R | 0.905 | Del |
| C49R | 0.711 | Del |
| F53C | 0.432 | Neu |
| E57K | 0.065 | Neu |
| V66A | 0.067 | Neu |
| **T71M** | **0.790** | **Del** |
| T82I | 0.314 | Neu |
| I85N | 0.512 | Prob. Del |
| **T86A** | **0.063** | **Neu** |
| F88C | 0.739 | Del |
| **F93V** | **0.671** | **Del** |
| F94S | 0.470 | Prob. Neu |
| L97I | 0.501 | Prob. Del |
| A103V | 0.311 | Neu |
| I105V | 0.047 | Neu |
| P110L | 0.128 | Neu |
| N117S | 0.220 | Neu |
| H118Y | 0.340 | Neu |
| H118R | 0.273 | Neu |
| **T119A** | **0.298** | **Neu** |
| **V122E** | **0.796** | **Del** |
| G127S | 0.300 | Neu |
| F132L | 0.621 | Del |
| C148Y | 0.450 | Prob. Neu |
| I157N | 0.660 | Del |
| L159M | 0.613 | Del |
| S164P | 0.613 | Del |
| V168I | 0.197 | Neu |
| I170V | 0.363 | Neu |
| M174I | 0.346 | Neu |
| M174V | 0.311 | Neu |
| R184S | 0.427 | Neu |
| **P185S** | **0.549** | **Prob. Del** |
| K186N | 0.660 | Del |
| R188H | 0.710 | Del |
| **R188C** | **0.671** | **Del** |
| T193M | 0.682 | Del |
| F194S | 0.835 | Del |
| K202E | 0.443 | Neu |
| R203W | 0.285 | Neu |
| R203P | 0.598 | Del |
| R203Q | 0.543 | Prob. Del |
| G205W | 0.790 | Del |
| L207I | 0.659 | Del |
| **L209F** | **0.801** | **Del** |
| I211L | 0.453 | Prob. Neu |
| R212Q | 0.767 | Del |
| R212P | 0.850 | Del |
| **A214V** | **0.742** | **Del** |
| A214G | 0.737 | Del |
| N215D | 0.707 | Del |
| **L220F** | **0.759** | **Del** |
| **G228E** | **0.930** | **Del** |
| L230P | 0.817 | Del |
| E240Q | 0.658 | Del |
| I247V | 0.058 | Neu |
| N250S | 0.339 | Neu |
| F251C | 0.854 | Del |
| D254H | 0.597 | Del |
| A255S | 0.206 | Neu |
| A255T | 0.212 | Neu |
| N257K | 0.110 | Neu |
| **P265L** | **0.864** | **Del** |
| H270Y | 0.693 | Del |
| I272V | 0.304 | Neu |
| F278S | 0.832 | Del |
| A283V | 0.247 | Neu |
| D290G | 0.659 | Del |
| T300A | 0.737 | Del |
| **T300I** | **0.641** | **Del** |
| S305C | 0.705 | Del |
| **R311Q** | **0.729** | **Del** |
| T312S | 0.721 | Del |
| P316L | 0.824 | Del |
| E318D | 0.586 | Del |
| V319L | 0.280 | Neu |
| **L320P** | **0.566** | **Del** |
| R324C | 0.402 | Neu |
| R324G | 0.323 | Neu |
| A326S | 0.544 | Prob. Del |
| I328T | 0.636 | Del |
| S330Y | 0.820 | Del |
| R338Q | 0.316 | Neu |
| N343K | 0.699 | Del |
| K346N | 0.755 | Del |
| T347R | 0.513 | Prob. Del |
| V350M | 0.665 | Del |
| T352S | 0.602 | Del |
| M357I | 0.267 | Neu |
| **M357T** | **0.298** | **Neu** |
| L359R | 0.467 | Prob. Neu |
| N361K | 0.567 | Del |
| K363T | 0.344 | Neu |
| D364H | 0.378 | Neu |
| D364G | 0.133 | Neu |
| V365I | - | - |
| A367D | - | - |
| K370E | - | - |
| Y373C | - | - |
| N377K | - | - |
| F378L | - | - |
| I379T | - | - |
| D387H | - | - |
| D387A | - | - |
| K390T | - | - |
| M391I | - | - |

## **S1 Table. Comprehensive list of ROMK missense mutations in the TOPMed database.**

Table shows the Rhapsody scores and predictions of 124 ROMK missense mutations from the TOPMed program that were analyzed in this study. The analysis was performed using a ROMK homology model that contains amino acids 38-364 (see **Fig 1**), so any residue outside of this range lacks a Rhapsody score and is designated “-“. “Del” indicates a substitution is predicted to be deleterious, whereas “Neu” (neutral) is predicted to have no effects on channel function. A designation of “Prob. Del” or “Prob. Neu” indicates that the Rhapsody score is close to the 0.5 cutoff for being deleterious. For example, the Rhapsody scores of I85N and F94S are 0.512 and 0.470, and thus, these mutations are categorized as “Prob. Del” and “Prob. Neu”, respectively. The mutations listed in **bold** were selected for growth analysis in yeast.
