# Supplementary material for: Genome mining yields putative disease-associated ROMK variants with distinct defects
Source: PLoS Genet. 2023 Nov 13;19(11):e1011051. doi: 10.1371/journal.pgen.1011051 (PMC10695394; doi:10.1371/journal.pgen.1011051)
Supplement: S2 Table — Rhapsody was used to predict ROMK mutation severity based on structural, evolutionary, and dynamic features. The analysis was performed with a tetrameric ROMK homology model (Uniprot number: P48048), which was built in Swiss-Model [148] based on the crystal structure of Kir2.2 (PDB ID: 3SPG). A Rhapsody pathogenicity probability (or “Rhapsody score”) was computed for each mutation, and a “Del” (deleterious) denotation was assigned if the probability is ≥ 0.5, whereas a “Neu” (neutral) indicates a probability of < 0.5. “Prob. Del” denotes that the Rhapsody probability is close to the 0.5 deleterious cutoff (i.e., P185S probability score is 0.549). * denotes an uncharacterized Bartter mutation, which was defined as a disease-associated mutation in ClinVar, but is listed as having uncertain clinical significance. ¶ denotes the mutation obtained from ClinVar. (DOCX) [file pgen.1011051.s010.docx]

| Mutation | Rhapsody score | Rhapsody prediction | Background information |
| --- | --- | --- | --- |
| **T71M*** | 0.790 | Del | Uncharacterized Bartter |
| **T86A** | 0.063 | Neu | Bartter |
| **F93V** | 0.671 | Del |  |
| **T119A*** | 0.298 | Neu | High Frequency, uncharacterized Bartter |
| **V122E** | 0.796 | Del | Bartter |
| **P185S** | 0.549 | Prob. Del | Bartter |
| **R188C*** | 0.671 | Del | May disrupt PIP_2_-dependent gating, uncharacterized Bartter |
| **L209F** | 0.801 | Del | Bartter |
| **A214V** | 0.742 | Del | May disrupt PIP_2_-dependent gating, Bartter |
| **L220F** | 0.759 | Del | May disrupt PIP_2_-dependent gating, Bartter |
| **G228E*** | 0.930 | Del | Uncharacterized Bartter,  high Rhapsody score |
| **P265L** | 0.864 | Del | High Rhapsody score |
| **T300I*** | 0.641 | Del | G-Loop, uncharacterized Bartter |
| **T300R***^,¶^ | 0.722 | Del | ClinVar, G-Loop, uncharacterized Bartter |
| **R311Q** | 0.729 | Del | Important for inter-monomeric interactions, Bartter |
| **L320P*** | 0.566 | Del | Uncharacterized Bartter |
| **M357T** | 0.298 | Neu | Highest Frequency |

## **S2 Table. Targeted list of 17 mutations showing Rhapsody scores, predicted phenotypes, and background information.**

Rhapsody was used to predict ROMK mutation severity based on structural, evolutionary, and dynamic features. The analysis was performed with a tetrameric ROMK homology model (Uniprot number: P48048), which was built in Swiss-Model (1) based on the crystal structure of Kir2.2 (PDB ID: 3SPG). A Rhapsody pathogenicity probability (or “Rhapsody score”) was computed for each mutation, and a “Del” (deleterious) denotation was assigned if the probability is ≥ 0.5, whereas a “Neu” (neutral) indicates a probability of < 0.5. “Prob. Del” denotes that the Rhapsody probability is close to the 0.5 deleterious cutoff (i.e., P185S probability score is 0.549). * denotes an uncharacterized Bartter mutation, which was defined as a disease-associated mutation in ClinVar, but is listed as having uncertain clinical significance. ¶ denotes the mutation obtained from ClinVar.

References

1. Bienert S, Waterhouse A, de Beer TA, Tauriello G, Studer G, Bordoli L, et al. The SWISS-MODEL Repository-new features and functionality. Nucleic Acids Res. 2017;45(D1):D313-D9.
