# Supplementary material for: Genome mining yields putative disease-associated ROMK variants with distinct defects
Source: PLoS Genet. 2023 Nov 13;19(11):e1011051. doi: 10.1371/journal.pgen.1011051 (PMC10695394; doi:10.1371/journal.pgen.1011051)
Supplement: S3 Table — The Rhapsody score (i.e., probability) and prediction for each of the 17 mutations, along with their growth phenotypes in medium supplemented with low potassium. For columns 4 and 5 (“ROMK), the growth assays of yeast expressing the mutations in the context of wild-type (WT) ROMK were conducted in 25mM KCl, and the “end-point” OD600 recordings at 48 hrs were normalized to WT. Each growth phenotype assessment shown in column 5 was based on the results of up to three independent experiments (representative graphs shown in Fig 2). The categorization of the growth defects was determined based on the normalized endpoint OD600 (to WT ROMK), and are defined as follows: No defect, OD ≥ 1 (1 = 100% WT); Slight defect, 0.9 ≤ OD < 1 (90–100% WT); Moderate defect: 0.8 ≤ OD < 0.9 (80–90% WT); Severe defect: OD < 0.8 (80% WT). The data in columns 6 and 7 (“ROMK-K80M”) represent growth phenotypes of yeast co-expressing the indicated allele and an activating mutation, K80M, in 10mM KCl medium (also see S1 Fig). The end-point OD600 values at 23.5 hrs and slopes of each of the growth curves (“Max V”, as calculated by the Gen5 software, BioTek Instruments, ver. 3.12) were normalized to the ROMK-K80M control. Data represent the means of 8 replicates. * denotes an uncharacterized Bartter mutation, and ¶ denotes a mutation in ClinVar. Additionally, a “-”marks where data were absent. (DOCX) [file pgen.1011051.s011.docx]

| **Name** | **Rhapsody score** | **Rhapsody prediction** | **ROMK (25mM KCl)** | | **ROMK-K80M (10mM KCl)** | |
| --- | --- | --- | --- | --- | --- | --- |
|  |  |  | **End-point OD_600_** | **Growth defect categorization** | **Endpoint OD_600_** | **Max V** |
| **Vector** | - | - | 0.60 | **-** | 0.18 | 0.11 |
| **ROMK** | - | - | 1.00 | **-** | 0.18 | 0.12 |
| **K80M** | 0.636 | Del | 2.39 | **-** | 1.00 | 1.00 |
| **Y314C** | 0.773 | Del | 0.88 | Moderate | 0.17 | 0.11 |
| **T71M*** | 0.79 | Del | 0.89 | Moderate | 0.71 | 0.66 |
| **T86A** | 0.063 | Neu | 0.96 | Slight | - | - |
| **F93V** | 0.671 | Del | 1.03 | None | **0.53** | **0.49** |
| **T119A*** | 0.298 | Neu | 1.03 | None | - | - |
| **V122E** | 0.796 | Del | 1.16 | None | **0.17** | **0.12** |
| **P185S** | 0.549 | Prob. Del | 1.10 | None | - | - |
| **R188C*** | 0.671 | Del | 1.12 | None | - | - |
| **L209F** | 0.801 | Del | 0.83 | Moderate | - | - |
| **A214V** | 0.742 | Del | 0.99 | Slight | - | - |
| **L220F** | 0.759 | Del | 0.82 | Moderate | - | - |
| **G228E*** | 0.93 | Del | 0.67 | Severe | 0.14 | 0.10 |
| **P265L** | 0.864 | Del | 0.95 | Slight | - | - |
| **T300I*** | 0.641 | Del | 1.05 | None | - | - |
| **T300R*^,¶^** | 0.722 | Del | 0.85 | Moderate | - | - |
| **R311Q** | 0.729 | Del | 0.81 | Moderate | 0.24 | 0.16 |
| **L320P*** | 0.566 | Del | 0.82 | Moderate | 0.18 | 0.12 |
| **M357T** | 0.298 | Neu | 1.24 | None | - | - |

## **S3 Table. Growth phenotype summary of yeast expressing TOPMed and ClinVar mutations.**

The Rhapsody score (i.e., probability) and prediction for each of the 17 mutations, along with their growth phenotypes in medium supplemented with low potassium. For columns 4 and 5 (“ROMK), the growth assays of yeast expressing the mutations in the context of wild-type (WT) ROMK were conducted in 25mM KCl, and the “end-point” OD_600_ recordings at 48 hrs were normalized to WT. Each growth phenotype assessment shown in column 5 was based on the results of up to three independent experiments (representative graphs shown in **Fig 2**). The categorization of the growth defects was determined based on the normalized endpoint OD_600_ (to WT ROMK), and are defined as follows: No defect, OD ≥ 1 (1 = 100% WT); Slight defect, 0.9 ≤ OD < 1 (90-100% WT); Moderate defect: 0.8 ≤ OD < 0.9 (80-90% WT); Severe defect: OD < 0.8 (80% WT). The data in columns 6 and 7 (“ROMK-K80M”) represent growth phenotypes of yeast co-expressing the indicated allele and an activating mutation, K80M, in 10mM KCl medium (also see **S1 Fig**). The end-point OD_600_ values at 23.5 hrs and slopes of each of the growth curves (“Max V”, as calculated by the Gen5 software, BioTek Instruments, ver. 3.12) were normalized to the ROMK-K80M control. Data represent the means of 8 replicates. * denotes an uncharacterized Bartter mutation, and ¶ denotes a mutation in ClinVar. Additionally, a “-“ marks where data were absent.
