# Supplementary material for: Genome mining yields putative disease-associated ROMK variants with distinct defects
Source: PLoS Genet. 2023 Nov 13;19(11):e1011051. doi: 10.1371/journal.pgen.1011051 (PMC10695394; doi:10.1371/journal.pgen.1011051)
Supplement: S6 Table — The top 5 rows of the table show the population distribution of binary disease phenotypes, i.e., “phecodes”, and the bottom 9 rows show the distribution of the metabolite disease phenotypes. “Hom.” denotes the number of individuals homozygous for the indicated mutation, “Het.” means heterozygous, and “WT” stands for wildtype, i.e., individuals without the indicated mutation. For the binary phenotypes, both the number of controls and cases are listed. (DOCX) [file pgen.1011051.s014.docx]

| **Phenotype** | **Chromosome position** | **Nucleotide change** | **Mutation** | **Controls** | | | **Cases** | | |
| --- | --- | --- | --- | --- | --- | --- | --- | --- | --- |
|  |  |  |  | Hom. | Het. | WT | Hom. | Het. | WT |
| Problems associated with amniotic cavity and membranes | 11:128839618 | C > T | G228E | 0 | 15 | 122037 | 0 | 2 | 532 |
| Hypertensive Heart Disease | 11:128839736 | C > T | A189T | 0 | 3 | 107271 | 0 | 1 | 167 |
| Hypopotassemia | 11:128839710 | G > A | N197N | 0 | 3 | 121124 | 0 | 1 | 531 |
| Electrolyte imbalance | 11:128839710 | G > A | N197N | 0 | 3 | 121124 | 0 | 1 | 564 |
| **Phenotype** | **Chromosome position** | **Nucleotide change** | **Mutation** | **Number of individuals** | | |  |  |  |
|  |  |  |  | Hom. | Het. | WT |  |  |  |
| Urea | 11:128839052 | T > A | 3’ UTR | 3206 | 33538 | 93583 |  |  |  |
| Phosphate | 11:128839052 | T > A | 3’ UTR | 2950 | 30896 | 85997 |  |  |  |
| Creatinine | 11:128839856 | C > A | V149L | 0 | 2 | 32081 |  |  |  |
| Urea | 11:128839856 | C > A | V149L | 0 | 12 | 131199 |  |  |  |
| Urea | 11:128839370 | G > A | R311W | 0 | 15 | 131197 |  |  |  |
| Sodium in urine | 11:128839736 | C > T | A189T | 0 | 4 | 133636 |  |  |  |
| Creatinine (enzymatic) in urine | 11:128839618 | C > T | G228E | 0 | 23 | 133898 |  |  |  |
| Systolic blood pressure (automated) | 11:128839170 | G > T | N377K | 0 | 27 | 129973 |  |  |  |
| Systolic blood pressure (manual) | 11:128839170 | G > T | N377K | 0 | 4 | 7800 |  |  |  |

## **S6 Table. Genotype distribution in the UK Biobank of ROMK variants with significant associations with disease phenotypes.**

The top 5 rows of the table show the population distribution of binary disease phenotypes, i.e., “phecodes”, and the bottom 9 rows show the distribution of the metabolite disease phenotypes. “Hom.” denotes the number of individuals homozygous for the indicated mutation, “Het.” means heterozygous, and “WT” stands for wildtype, i.e., individuals without the indicated mutation. For the binary phenotypes, both the number of controls and cases are listed.
