# Supplementary material for: Genome mining yields putative disease-associated ROMK variants with distinct defects
Source: PLoS Genet. 2023 Nov 13;19(11):e1011051. doi: 10.1371/journal.pgen.1011051 (PMC10695394; doi:10.1371/journal.pgen.1011051)
Supplement: S7 Table — Table shows the pathogenicity predictions made by the indicated computational tools for the 17 TOPMed and ClinVar mutations. The second column shows the phenotype exhibited by each mutant when expressed in yeast, as shown in S3 Table. The growth phenotype noted with an asterisk (*) indicates that a growth defect was only observed when the mutant was expressed in the context of the K80M allele (S1 Fig). In the remaining columns, a mutation that is predicted to be pathogenic is marked with “Del” (deleterious), while a benign mutation is designated “Neu” (neutral). Whether there was an uncertainty in the prediction, or a prediction is unavailable, is also indicated (“Uncertain” or “N/A”). In the bottom row, an accuracy assessment for each method based on yeast growth phenotype is provided. A “slight” growth defect was counted for both deleterious and neutral. The computational tools employed are Rhapsody [40], Polyphen-2 [41], Evmutation [42], EVE [125], SNPs&GO [161]. (DOCX) [file pgen.1011051.s015.docx]

| Mutation | Growth phenotype | Rhapsody | PolyPhen-2 | EVmutation | EVE | SNPs&GO |
| --- | --- | --- | --- | --- | --- | --- |
| T71M | Moderate | Del | Del | Del | Del | Del |
| T86A | Slight | Neu | Neu | Neu | Neu | Neu |
| F93V | None* | Del | Del | Neu | Neu | Del |
| T119A | None | Neu | Del | Neu | Neu | Neu |
| V122E | None* | Del | Del | Del | Del | Del |
| P185S | None | Prob. Del | Neu | Neu | Uncertain | Del |
| R188C | None | Del | Del | Del | Del | Del |
| L209F | Moderate | Del | Del | Neu | Uncertain | Del |
| A214V | Slight | Del | Del | Del | Uncertain | Del |
| L220F | Moderate | Del | Del | Del | Uncertain | Del |
| G228E | Severe | Del | Del | Del | Del | Del |
| P265L | Slight | Del | Del | Del | Del | Del |
| T300I | None | Del | Del | Neu | Neu | Neu |
| T300R | Moderate | Del | Del | Del | Del | Del |
| R311Q | Moderate | Del | Del | Del | Del | Del |
| L320P | Moderate | Del | Del | Del | Del | Del |
| M357T | None | Neu | Neu | N/A | Neu | Neu |
| Accuracy |  | 14/17 | 14/17 | 13/17 | 11/17 | 15/17 |

## **S7 Table. Pathogenicity predictions of 17 TOPMed and ClinVar mutations made by different computational methods.**

Table shows the pathogenicity predictions made by the indicated computational tools for the 17 TOPMed and ClinVar mutations. The second column shows the phenotype exhibited by each mutant when expressed in yeast, as shown in **S3 Table**. The growth phenotype noted with an asterisk (*) indicates that a growth defect was only observed when the mutant was expressed in the context of the K80M allele (**S1 Fig**). In the remaining columns, a mutation that is predicted to be pathogenic is marked with “Del” (deleterious), while a benign mutation is designated “Neu” (neutral). Whether there was an uncertainty in the prediction, or a prediction is unavailable, is also indicated (“Uncertain” or “N/A”). In the bottom row, an accuracy assessment for each method based on yeast growth phenotype is provided. A “slight” growth defect was counted for both deleterious and neutral. The computational tools employed are Rhapsody (1), Polyphen-2 (2), Evmutation (3), EVE (4), SNPs&GO (5)

References

1. Ponzoni L, Penaherrera DA, Oltvai ZN, Bahar I. Rhapsody: Predicting the pathogenicity of human missense variants. Bioinformatics. 2020.

2. Adzhubei I, Jordan DM, Sunyaev SR. Predicting functional effect of human missense mutations using PolyPhen-2. Curr Protoc Hum Genet. 2013;Chapter 7:Unit7 20.

3. Hopf TA, Ingraham JB, Poelwijk FJ, Scharfe CP, Springer M, Sander C, et al. Mutation effects predicted from sequence co-variation. Nat Biotechnol. 2017;35(2):128-35.

4. Frazer J, Notin P, Dias M, Gomez A, Min JK, Brock K, et al. Disease variant prediction with deep generative models of evolutionary data. Nature. 2021;599(7883):91-5.

5. Calabrese R, Capriotti E, Fariselli P, Martelli PL, Casadio R. Functional annotations improve the predictive score of human disease-related mutations in proteins. Hum Mutat. 2009;30(8):1237-44.
