# Supplementary material for: Genome mining yields putative disease-associated ROMK variants with distinct defects
Source: PLoS Genet. 2023 Nov 13;19(11):e1011051. doi: 10.1371/journal.pgen.1011051 (PMC10695394; doi:10.1371/journal.pgen.1011051)
Supplement: S8 Table — (DOCX) [file pgen.1011051.s016.docx]

| **Primer name** | **Sequence (5’ to 3’)** |
| --- | --- |
| **T71MF** | CATCTGGACAATGGTGCTGGACC |
| **T71MR** | GGTCCAGCACCATTGTCCAGATG |
| **T86AF** | CGTGTTCATCGCAGCCTTCTTG |
| **T86AR** | CAAGAAGGCTGCGATGAACACG |
| **F93VF** | GGGGAGTTGGGTTCTCTTTGGTC |
| **F93VR** | GACCAAAGAGAACCCAACTCCCC |
| **T119AF** | CTGACAACCGCGCTCCTTGTGTG |
| **T119AR** | CACACAAGGAGCGCGGTTGTCAG |
| **V122EF** | CGCACTCCTTGTGAGGAGAACATTAATG |
| **V122ER** | CATTAATGTTCTCCTCACAAGGAGTGCG |
| **R185SF** | GATCTCTAGATCGAAAAAACGTGC |
| **R185SR** | GCACGTTTTTTCGATCTAGAGATC |
| **R188CF** | GACCCAAAAAATGTGCTAAAAC |
| **R188CR** | GTTTTAGCACATTTTTTGGGTC |
| **L209FF** | GAAGCTCTGCTTCCTCATCCG |
| **L209FR** | CGGATGAGGAAGCAGAGCTTC |
| **A214VF** | CATCCGAGTGGTAAATCTTAGGAAG |
| **A214VR** | CTTCCTAAGATTTACCACTCGGATG |
| **L220FF** | CTTAGGAAGAGCTTCCTGATTGGCAG |
| **L220FR** | CTGCCAATCAGGAAGCTCTTCCTAAG |
| **G228EF** | CAGCCACATATATGAGAAGCTTCTAAAGAC |
| **G228ER** | GTCTTTAGAAGCTTCTCATATATGTGGCTG |
| **P265LF** | CTTCATATCCCTACTGACGATC |
| **P265LR** | GATCGTCAGTAGGGATATGAAG |
| **T300IF** | CTTTTTAGATGGCATAGTGGAATCCACC |
| **T300IR** | GGTGGATTCCACTATGCCATCTAAAAAG |
| **T300RF** | CTTTTTAGATGGCAGAGTGGAATCCACC |
| **T300RR** | GGTGGATTCCACTCTGCCATCTAAAAA |
| **R311QF** | CTGCCAGGTCCAAACGTCATACG |
| **R311QR** | CGTATGACGTTTGGACCTGGCAG |
| **L320PF** | GTCCCAGAGGAGGTGCCTTGGGGCTACCGTTTC |
| **L320PR** | GAAACGGTAGCCCCAAGGCACCTCCTCTGGGAC |
| **M357TF** | CTCACTGTGCCACTTGCCTCTATAATG |
| **M357TR** | CATTATAGAGGCAAGTGGCACAGTGAG |
| **Y314C_f** | GTCCGCACGTCATGCGTCCCAGAGGAG |
| **Y314C_r** | CTCCTCTGGGACGCATGACGTGCGGAC |
| **V149L_UKBB_QC_fwd** | ATAGGTTACGGATTCAGGTTTTTGACAGAACAGTGCG |
| **V149L_UKBB_QC_rev** | CGCACTGTTCTGTCAAAAACCTGAATCCGTAACCTAT |
| **R311W_UKBB_QC_fwd** | CAACCTGCCAGGTCTGGACGTCATACGTCCC |
| **R311W_UKBB_QC_rev** | GGGACGTATGACGTCCAGACCTGGCAGGTTG |
| **A189T_UKBB_QC_fwd** | GATCTCTAGACCCAAAAAACGTACCAAAACCATTACGTTCAGCAAGA |
| **A189T_UKBB_QC_rev** | TCTTGCTGAACGTAATGGTTTTGGTACGTTTTTTGGGTCTAGAGATC |
| **N377K_UKBB_QC_fwd** | AGAGGCTATGACAACCCTAAATTTGTCTTGTCAGAAGTTG |
| **N377K_UKBB_QC_rev** | CAACTTCTGACAAGACAAATTTAGGGTTGTCATAGCCTCT |
| **Antibody** (Dilution) | **Information** |
| **ROMK** (1:1000-2000) | Rabbit antiserum from the Welling lab, Baltimore, MD, USA (1). |
| **G6PD** (1:5000) | Rabbit polyclonal, from Sigma-Aldrich, St. Louis, MO, USA (A9521). |
| **Hsp90** (1:1000) | Mouse monoclonal, from Enzo Life Sciences, Farmingdale, NY, USA (ADI-SPA-830-D). |
| **Na^+^/K^+^-ATPase** (1:1000) | Mouse monoclonal, from Developmental Studies Hybridoma Bank, Iowa City, IA, USA (a5). |
| **β-Actin** (1:5000) | Mouse monoclonal, from Abcam, Cambridge, UK (ab6276). |
| **Rabbit** (1:5000) | Goat, horseradish peroxidase (HRP)-conjugated, from Cell Signaling Technology, Danvers, MA, USA (7074S). |
| **Mouse** (1:5000) | Horse, horseradish peroxidase (HRP)-conjugated, from Cell Signaling Technology, Danvers, MA, USA (7076S). |
| **Yeast strain** | **Genotype (Origin)** |
| ***trk1*Δ*trk2*Δ** | *MATα his3*Δ *leu2*Δ *ura3*Δ *trk1*Δ*::URA3 trk2*Δ*::NATMX can1*Δ*::STE2pr-HIS3* (2). |
| ***pdr5*Δ** | *MATα, his3*Δ, *leu2*Δ, *ura3*Δ, *pdr5::KANMX* (Invitrogen, Waltham, MA, USA). |
| **BY4742** | *MATα his3*Δ*, leu2*Δ*, ura3*Δ (Invitrogen, Waltham, MA, USA). |
| ***cdc48-2*** | *MATα his3*Δ, *leu2*Δ, *ura3*Δ, *cdc48-2::KANMX* (3). |

## **S8 Table. Primers, yeast strains, and antibodies used in this study.**

References

1. Wade JB, Fang L, Coleman RA, Liu J, Grimm PR, Wang T, et al. Differential regulation of ROMK (Kir1.1) in distal nephron segments by dietary potassium. Am J Physiol Renal Physiol. 2011;300(6):F1385-93.

2. Kolb AR, Needham PG, Rothenberg C, Guerriero CJ, Welling PA, Brodsky JL. ESCRT regulates surface expression of the Kir2.1 potassium channel. Mol Biol Cell. 2014;25(2):276-89.

3. Moir D, Stewart SE, Osmond BC, Botstein D. Cold-sensitive cell-division-cycle mutants of yeast: isolation, properties, and pseudoreversion studies. Genetics. 1982;100(4):547-63.
