# Supplementary material for: Genome mining yields putative disease-associated ROMK variants with distinct defects
Source: PLoS Genet. 2023 Nov 13;19(11):e1011051. doi: 10.1371/journal.pgen.1011051 (PMC10695394; doi:10.1371/journal.pgen.1011051)
Supplement: Attachment — Submitted filename: Response to reviewers comments.docx [file pgen.1011051.s017.docx]

We sincerely thank the editor and the reviewers for taking the time to read and critique our manuscript. We have taken the comments to heart and have made considerable efforts to revise the manuscript. Some notable changes are: Three new figures (S1 Figure, S6 Figure, and S8 Figure), two new tables (S5 Table and S7 Table), and significant changes to a figure and a table (Figure 2 and S3 Table). These many modifications result from performing four additional experiments and analysis:

- Yeast growth assays in the context of a hyperactive (K80M) allele (S2 Figure and S3 Table).
- ROC curve analysis to assess the predictive power of Rhapsody (S6 Figure).
- Whole-cell current measurements in *X. laevis* oocytes to assess mutant heterozygosity (S8 Figure).
- Pathogenicity prediction comparison of different computational tools (S7 Table).

We also went over the manuscript line-by-line and made numerous changes to the text and the figures/tables to improve clarity and flow of the text, and to better highlight the goals and findings of our study. Some of these changes also directly address other reviewer concerns.

Below please find our detailed response to the points raised by each reviewer.

**Reviewer #1**: In “Genome mining yields new disease-associated ROMK variants with distinct defects”, Nguyen et al describe a combined computational and experimental approach to investigate variants of the ROMK ion channel, which are associated with Bartter syndrome.

Computational methods have long been used to predict loss-of-stability variants that are likely degraded by the proteasome (Pey et al. 2007). Recently a method optimised for membrane proteins - which require special handling due to the different environments of the transmembrane and the intra-&extracellular elements - has become available (Tiemann et al. 2023). It is, however, not clear whether the tool employed by the authors (Rhapsody) is suitable for identification of loss-of-stability variants specifically, or if it is a more general loss-of-function predictor like GEMME or EVE. As shown in above-referenced paper, those approaches can be combined favorably to distinguish which variants are detrimental because of loss of stability, and which are detrimental for other reasons such as loss of binding, catalytic activity, ion conductivity, or other relevant functions.

If the authors think that Rhapsody is suited for identifying destabilised variants, they need to substantiate this. Otherwise they may want to consider including a more biophysics-based method that aims at predicting stability changes. This may also help with the fact that in the current manuscript, only 10/16 of the selected variants actually show increased proteasomal degradation experimentally.

We apologize that we had not thoroughly outlined the value of Rhapsody compared to other predictive programs. Therefore, we added a paragraph in the Results (lines 161-173) and a paragraph in the Discussion (lines 595-623) to expand on previously published accuracy measurements done for Rhapsody, as well as to clarify that we did not intend to use Rhapsody to search for only destabilized/unstable variants. Rather, the goal was to more generally identify deleterious variants. We also computed a new ROC curve for Rhapsody based on the yeast growth assay data (S6 Fig) and provided a comparison chart for the pathogenicity prediction between different computational tools (S7 Table). Combined with new growth assay data (S1 Fig), we now find 17 out of 21 variants exhibited growth phenotypes in accordance with their Rhapsody predictions.

Once this issue is addressed it is a promising study, membrane proteins are enriched in pathogenic variants but at the same time challenging for many experimental and computational methods, so this integrated method and experimental follow-up may well be interesting beyond ROMK and Bartter syndrome.

**Minor comments:**

In the abstract, it does not become clear what the broader goal of the study is (early diagnosis? Genetic counseling? Treatment?). The Author Summary mentions treatment options, if these are the main motivation this should also be mentioned in the abstract, and perhaps illustrated with examples in the introduction.

We thank the reviewer for pointing this out – we have revised the abstract and added several sentences at the end of the discussion to underscore the goal of our study. In short, our goal is to advance precision medicine and the development of targeted treatment options for patients with different disease-causing alleles.

The authors state in the introduction that their screening method is suitable for high-throughput application, but then proceed to test 17 variants, which by most people in the community would not be considered high throughput. If the authors wish to discuss the high-throughput detection of destabilising variants, they should include a brief discussion of similar methods like VAMP-seq (Chiasson et al. 2020).

We apologize for this oversight in the first draft of our manuscript. We have now included a brief discussion of high-throughput methods, such as VAMP-seq and deep mutational scanning, in the Discussion. We also now refrain from using the term “high-throughput” when referring to the nature of our study. While the yeast system is powerful and can be scaled up to test hundreds of variants at once, we characterized a smaller number of variants as a proof-of-concept for our pipeline in this study. In addition, since Rhapsody can provide predictions for all possible amino acid substitutions available in protein for which a homology model can be generated, we deposited a comprehensive list of all these predictions on GitHub (please see the link in the Materials & Methods).

As for the computational parts of the method, it could be nice to apply it to the entire protein and hence cover all possible variants - some of which may be detected in future human genome/exome sequencing.

In addition to the comprehensive list of all Rhapsody predictions for the entire protein, which was deposited on GitHub (please see the link in the Materials & Methods), this file also includes predictions from PolyPhen-2 and EVmutation.

In terms of what Rhapsody scores make a good cutoff, the authors could perform a ROC analysis.

We thank the reviewer for this excellent suggestion. To this end, we have performed the requested ROC analysis and the result of this analysis is outlined in S6 Fig. In brief, the value obtained was 0.76 when the True Positive Rate versus the False Positive Rate were compared and analyzed.

**Reviewer #2**

In their manuscript entitled “Genome mining yields new disease-associated ROMK variants with distinct defects”, Nguyen et al report their workflow for identifying mutations in the ROMK potassium channel associated with clinical symptoms such as Bartter syndrome. Guided by computational tools, this study presents a systematic approach for characterizing mutant variants of ROMK in human populations that may confer disease phenotypes. First, the authors mine three different databases for ROMK mutant variants associated with clinical features. They then prioritize these mutations based on computational prediction of the impact of the substitution (Rhapsody). The study follows up on prioritized mutations using a powerful yeast model system to assay surface potassium transport function of ROMK variants. They also use the yeast model and cultured HEK293 cells to assess how mutations affect protein degradation and stability. Finally, for a mutation that was stable but not functional (T300R), the authors examine channel activity in Xenopus oocytes and determine this mutant variant is defective for channel activity. Together, this study presents a strategy for mining and characterizing disease mutations, which will be of interest to those studying ROMK and Bartter syndrome and also to researchers interested in mining and characterizing disease mutations in general, particularly for ion channels. Overall, the manuscript is well-written, and the authors provide a balanced discussion of the advantages and limitations of their approach. Here are a few comments and suggestions for strengthening the manuscript:

**Major Points:**

1. Regarding Figure 2 and Figures S1 and S2: As presented, there is not much dynamic range provided by the yeast plating assay. Indeed, none of the results shown in 2B reveal a compelling growth defect, and the two examples marked by black triangles look very similar. The liquid culture growth assays provide more compelling evidence of a growth defect with a clear dynamic range of phenotypes. However, the quantitative criteria the authors used to bin mutant variants into phenotypic categories (severe, moderate, and slight growth defects) was not clearly articulated. It would be helpful if these criteria were defined by a specific quantitative standard. (e.g., does “severe” mean statistically indistinguishable from vector only? And for some of the “slight” defect mutants, they look essentially like WT ROMK. What significance thresholds are being used?).

We thank the reviewer for highlighting this concern, and upon further reflection we completely agree. In brief, we too realize the shortcomings of the plate assays and have thus removed these data from the manuscript, leaving only the more quantitative liquid culture growth assays. Moreover, to assess the results of the growth assay in a more quantitative manner, we expanded S3 Table to include the calculated end-point OD readings (i.e., the OD of yeast at the end of the growth assay) and V max values. Overall, the results from this more quantitative analysis support our prior conclusions and can more readily be appreciated.

2. The N377K mutant variant of ROMK is fascinating because it functions like WT ROMK in the yeast system despite its clear loss of stability. Based on the data in Figure 5, inhibiting the proteasome restores some but not all stability – suggesting that a population of N377K ROMK escapes ERAD and is degraded by another mechanism. The surface labeling experiment shown in Figure S5 indicates that, despite its lower expression level, it is present at the cell surface. (Indeed, it appears that the percentage of N377K that makes it to the surface may be higher than any other mutant, based on the blot shown in Figure S5.) How such low surface levels of N377K ROMK provide potassium transport activity equivalent to WT ROMK is not clear, but it seems to suggest that this mutant has enhanced channel activity, despite its decreased stability at the level of the ER. Can this hypothesis be tested using the Xenopus oocyte system to analyze channel activity of the N377K mutant?

The reviewer makes a terrific suggestion. Indeed, the phenotypes associated with this variant are exceptionally intriguing. As suggested, we have now performed TEVC experiments (S8 Fig) and found that oocytes expressing the N377K mutant might exhibit a small albeit not statistically significant reduction in whole-cell currents, compared to the currents exhibited by wild-type channels. Combined with other the other phenomena outlined in the text, this added effect provides yet another explanation for the results from the yeast growth assays. A discussion of this mutation has consequently been expanded.

3. It is noteworthy that some mutants (F93V, V122E, and others) were predicted to be deleterious by Rhapsody but exhibited no defect in yeast growth assays. The authors don’t characterize this category of mutant but given the results of the N377K mutant (low stability, normal function) it seems plausible that these mutants could exhibit stability/degradation phenotypes but normal function – which would potentially put these mutants in the same category as N377K. Therefore, the authors should include analysis of these mutants (or at least one or two representatives) in their stability assays. Regardless of the outcome, adding this analysis will improve understanding of their pipeline and may even provide interesting new results.

As suggested by the reviewer, we chose select representative variants, i.e., F93V and V122E, to perform growth assays in the context of a hyperactive allele, K80M, in yeast. Both mutants exhibited considerable growth defects in the context of the K80M mutant. Consequently, the lack of a growth defect observed previously might have been attributed to the signal-to-noise (also see comment #1, above).

4. Line 487-489: The authors suggest a correlation between Rhapsody score and degradation/stability. It would be interesting to present this correlation for each of the mutants tested, in order to determine the extent of correlation between Rhapsody score and degradation/stability. This could be added with the data on hand, and it could provide insights into how good Rhapsody is at predicting stability (compared to yeast growth assays, for example).

We agree that it is impossible to use Rhapsody to predict mutant protein stability, and we thank the reviewer for this comment. Instead, Rhapsody is a powerful pathogenicity prediction tool, but as pointed our elsewhere and in the revised text, pathogenicity does not necessarily correlate with stability. Indeed, our new TEV data support this view. As a result, the text has been modified throughout to alter our previous erroneous conclusion, and text has been added in the Discussion to highlight this point.

**Minor Points:**

1. Table S3 provides a lot of value for the paper, but the qualitative nature of the table detracts from the value somewhat. Can this table be revised to incorporate more quantitative metrics of the phenotypes being reported? (e.g., Rhapsody score and more quantitative growth metrics).

We thank the reviewer again for this suggestion, and we have added other quantitative metrics to this table. (Also see Major Point 1, above.)

2. In immunoblots for analysis of protein stability (Figure 5 and Figure 6) quantification is presented as a graph of “% remaining”. However, in most of the immunoblots shown it is not clear what in the blot is being quantified. In the figure legend, the authors should indicate the band being quantified.

We apologize for the oversight and have now amended the legends of these figures to clarify which bands in the immunoblots were quantified.

3. Similarly, for the immunoblots shown in Figure 7A and quantified in 7B, it is unclear which band on the blot is being quantified.

We have also amended the legend of this figure to clarify which bands in the immunoblots were quantified.

4. In the discussion, the authors comment on the narrow dynamic range of the yeast assays for ROMK. I wonder if the K80M variant of ROMK be used to enhance the dynamic range of the yeast plating assay and/or liquid culture assay. Combining K80M with other mutations to be characterized might provide a more sensitive readout for the function of mutants with slight defects or reveal defects in mutations that are otherwise wildtype in the yeast assays as presented.

The reason why we initially did not perform growth assays in the context of a hyperactive variant was because complex intramolecular interactions between a given loss-of-function mutation and a hyperactive mutation—which likely exhibits allosteric and not structural effects—might exist. Regardless, we acknowledge and comment more clearly on the small dynamic range of the ROMK yeast growth assays and therefore selected representative mutations to perform growth assays in the context of the K80M hyperactive variant (S1 Fig and S3 Table). Two deleterious variants (F93V and V122E) that were without defects (likely because of a marginal signal-to-noise) can now be seen to exhibit measurable growth defects in the context with K80M. Together, these results suggest that adding analyses in the context of a hyperactive allele will prove valuable in future efforts.

5. One impactful aspect of this manuscript is the insights it provides for how predictive the computational tools (specifically, Rhapsody) are for identifying mutants with defects in stability and function. The data presented in this study provide some new insights into the strengths and weaknesses of this approach, and if specific predictions are high or low confidence. This is addressed somewhat in the discussion (line 570-571, line 604-606). However, I think the discussion would be strengthened if the authors included a section/paragraph that iterates the features of a high-confidence prediction and contrasts that to the features of a low-confidence prediction. This will help readers to better understand how to interpret the data presented in some of the supplemental tables, or other studies using Rhapsody.

We have included a ROC analysis to assess the accuracy of Rhapsody (S6 Fig), as well as a table comparing Rhapsody predictions with those from other pathogenicity prediction tools (S7 Table). When combined with the more quantitative assessment of the growth assays, we are confident that these additions will better inform readers who wish to employ our pipeline for future studies.

**Reviewer #3**: This is an ambitious paper that seeks to identify rare disease causing ROMK (KCNJ1) variants by means of database mining, functional analyses, and correlation with human phenotypes.

Certainly, they have identified a number of rare missense variants in ROMK and characterized these through a set of physiologic assays.  This work appears to be thorough and comprehensive, looking at the encoded proteins electrophysiologically, by assays of membrane localization, ERAD, etc.

My concern with this manuscript relates to the analyses connecting these variants with human phenotypic data.  Many of the phenotypes chosen to look at are pretty common and none are at all specific for Bartter’s syndrome (Tables 1, 2).  It is pretty hard to know what a significant p value should be here and how to properly correct for multiple hypotheses (or the ‘phenome”?).  Some of the details of choices of phenotypes seem peculiar to me. Why is “hypopotassemia” (meaning hypokalemia I assume) a binary trait (Table 1) but urea, phosphate, etc not treated as binary?

We thank the reviewer for bringing up this issue. Thanks to this comment, we have now made significant changes to the text and the tables to include the list of phenotypes analyzed, as well as to clarify the pipeline for the association analyses we performed. To consult the full list of phenotypes analyzed in our study, please refer to S5 Table. We also amended the legends of Tables 1 and 2 to explain the differences between a binary trait versus a quantitative phenotype. Specifically, we defined a binary trait as a trait that can only be present or absent in an individual. One example for such trait would be “electrolyte imbalance”. On the other hand, phenotypes such as urea and phosphate can be measured and compared between wild-type and heterozygous individuals, and are thus treated as non-binary.

We used the term “hypopotassemia” because this is the term listed in the available clinical ICD-10 phenotypes.

We recognize the difficulty in pinpointing a significant p value as well as finding true disease associations with a variant, as the reviewer rightly points out. This is especially true given the relatively low number of individuals carrying these mutations, which is of course typical for rare recessive diseases. We therefore agree that follow-up functional assays are crucial in determining whether a variant is potentially disease-associated, as now highlighted in the revised text. More generally, as outlined below, we have softened any claims that the identified and characterized alleles are linked to disease, but instead they are potentially associated with disease. The title of the manuscript has also been changed.

Why is alkalosis (or elevated sodium bicarbonate or high urine pH) not included among the various Bartter syndrome phenotypes?

We apologize for not including a list of all phenotypes analyzed in the original manuscript. This did include alkalosis. A new table, S5 Table, has been added to the revised manuscript and the second tab shows alkalosis.

It appears that all of these rare variants are ultimately “loss-of-function” in effect. The fact that different variants are associated with different phenotypes makes me think these are likely spurious associations. I would expect overlap in phenotypes if these are real associations. And adds complexity to the analysis of statistical significance.

As noted above, we agree with the limitations of the study, and now better outline the limitations of using current databases. Yet, as these expand over time, potentially spurious associations should be more readily identified. We also better highlight the fact that the individuals with loss-of-function alleles are complex heterozygotes, and new experiments to analyze potentially complex phenotypes have been added (see comment below). Finally, we have softened any claims that the identified mutations are linked to disease, and instead note that these are putative disease-associated alleles. As a result, the title has also been changed.

Some oddities may reflect the very small numbers (SBP  manual versus automated in Table 3 in people with mutation - very different, but not in controls.)

We completely agree with this assessment and have added text to the revised manuscript to again highlight the small numbers that can currently be analyzed. It is also worth noting—as we added to the Discussion and noted above—that over time, the databases will continue to grow so improved data/conclusions on disease association should become available.

In Table 2, why is the N377K variant labeled as “synonymous”?

We thank the reviewer for pointing this out. This was a typographical error and has now been amended.

In Table 3, why is the N377K found in 4 people in row 8 but 27 people in row 9?

We apologize for the confusion. We have therefore editing Tables 1 – 3 to improve clarity. In short, the data for manual measurements of systolic pressure are available for four individuals carrying the N377K mutation (row 8, Table 3), whereas automated measurement data are available for 27 individuals with this mutation (row 9, Table 3).

Certainly, it is quite likely that these variants would be disease causing if inherited with another similar mutation on the second allele. This is pretty typical for recessive diseases.  But the connection of the heterozygous state to these phenotypes seems likely unrelated to any change in ROMK function.

We thank the reviewer for this comment. We acknowledge that heterozygosity is very common in rare recessive diseases, and the phenotypic presentations of heterozygous carriers are often unclear. To this end, we expressed two representative disease-associated mutations from our study, G228E and T300R, and asked if heterozygous expression of these alleles would result in any effects. We found that whole-cell currents in *X. laevis* oocytes expressing half of the wild-type channel and half of the mutant are approximately 50% of the wild-type currents (S8 Fig), which we think are consistent with our data that these individuals might exhibit some mild phenotypes related to Bartter syndrome. Perhaps the intermediate phenotypes exhibited by these individuals provide us with an explanation for why we don’t see a lot of overlapping phenotypes across the variants.
