# Supplementary material for: Genome mining yields putative disease-associated ROMK variants with distinct defects
Source: PLoS Genet. 2023 Nov 13;19(11):e1011051. doi: 10.1371/journal.pgen.1011051 (PMC10695394; doi:10.1371/journal.pgen.1011051)
Supplement: Attachment — Submitted filename: Response to reviewers comments.docx [file pgen.1011051.s018.docx]

**Response to reviewers’ comments**

We thank the editor and all three reviewers for taking the time to reevaluate our revised manuscript and for providing valuable feedback to help us further improve this paper. In brief, we have made numerous changes to the text to address their additional comments. In addition, a significantly expanded version of **S6 Fig** are included to further assess the accuracy of Rhapsody compared to other algorithms. An accompanying table to this was also uploaded to Github to detail how we computed the ROC curves. Minor changes were made to **Fig 2**, as well as to the legends of **Fig 2** and **S3 Table**. More detailed responses are provided below.

**Reviewer #1**

*In their revised manuscript, the authors have made several additions and revisions that have significantly strengthened the study. For example, the addition of new experiments analyzing mutations in the context of the hyperactive K80M allele to enhance the dynamic range of the growth phenotype serves to strengthen the manuscript. Overall, this is an excellent study that is well-written and will appeal to the readership of PLOS Genetics. I have only a couple of very minor comments on the revised manuscript:*

*Minor point*

*1. Inclusion of the end point OD600 in Table S3 is very helpful. However, it is still unclear to me what thresholds are being used for each growth defect category, particularly in the case of the “slight defect” mutants. For example, A214V has an end point OD of 0.99, compared to 1.00 for the ROMK positive control. Does this really constitute a “slight difference”? I still can’t find a clear description of how mutants were categorized in the main text or in the legend of either Figure 2 or Table S3. It looks like it was all based on end point OD, with a moderate defect corresponding to 80-90% of WT, while a slight defect is 90-99% of WT. Just clarifying this in the text or one of the legends would be very helpful.*

Based on this comment, we have performed a more quantitative assessment of yeast growth defects based on the relative “endpoint” for yeast growth, i.e., optical density (OD) values at a time point that best reflects the diverse growth effects observed. The growth defect categories are in accordance with the reviewer’s suggestion above, and are now defined in better detail in the legends of **Fig 2** and **S3 Table**. In short, we chose the break-points for each categorization as follows: No defect, OD ≥ 1 (or 100% WT); Slight defect, 0.9 ≤ OD < 1 (90-100% WT); Moderate defect: 0.8 ≤ OD < 0.9 (80-90% WT); Severe defect: OD < 0.8 (80% WT). Based on this assessment, we moved T71M (with OD_600_ of 0.89, or 89% of wild-type) from the “Slight defect” to the “Moderate defect” category in **Fig 2**, and revised the figure to reflect this change. It is also important to note that the break-points between the categories were now chosen in part since there were clear differences between the ODs in distinct categories. This is also spelled-out in the Results section.

*Minor edits*

*1. Lines 76-79: this sentence is repetitive and needs to be revised.*

*2. Line 442: I think “datamining” should be two words*

We have made the requested edits to the text to address both comments above.

**Reviewer #2**

*In "Genome mining yields putative disease-associated ROMK variants with distinct defects", Nguyen et al. present a revised version of a previous manuscript on the same topic. The authors have extensively commented on the reviewer comments and clearly put in a lot of additional work to improve the manuscript. Below are some comments that would be good to clarify for a final version of the article.

1. The abstract mentions "a computational algorithm that predicts protein misfolding and disease severity" - it would be good to name the algorithm, probably Rhapsody? Also, is it really misfolding (following up on the first round of reviews, where this question also came up) that is assessed? The term keeps popping up throughout the manuscript although the response to reviewers mentions that indeed Rhapsody is not folding-specific, so it's unclear if additional edits are missing here.*

*2. Overall, it should be clear throughout the manuscript when protein misfolding is meant (e.g. from prior work focusing on that) and when general pathogenicity prediction or assessment of function is carried out.*

Combined response for points 1 and 2:

We thank the reviewer for bringing the noted inconsistency and need for better clarification to our attention. Rhapsody provides a pathogenicity probability (between 0 and 1) that determines whether a mutation is likely pathogenic or benign, but the reviewer is absolutely correct that the algorithm does not specifically assess misfolding. We apologize for using this term on occasion in prior versions of the text, but this has now been changed (e.g., in the Abstract, as requested). We also believe the developers of Rhapsody did not intend to restrict the algorithm to only mutations that lead to protein misfolding. Details are provided in two papers reporting on Rhapsody that are cited in the manuscript. However, since Rhapsody incorporates structural and dynamical features in its prediction, some mutations that compromise structural integrity, which encompass misfolding variants, are predicted to be deleterious. We also now make this point clear in the manuscript.

The goal of our study was to perform preliminary assessments of ROMK human variants using yeast growth assays, followed by further functional analysis, with a focus on finding new variants that compromise folding. We therefore used Rhapsody to guide our variant selection for the initial yeast growth assessment and then assessed misfolding with the follow-up assays downstream of the pipeline. While we realized could have used a more “folding-specific” predictive algorithm, we also did not want to exclude new variants that are channel-defective, such as T300R, which is characterized in this report. Ultimately, our revised discussion of Rhapsody in the **Discussion** section should rectify this concern.

3. It should further be clarified if Rhapsody assesses disease severity - via a quantitative score - or is mainly a binary assessment of whether a variant is pathogenic or not, which is how it appears to be used in Table S7.

Rhapsody provides predictions in the form of numerical scores (i.e., pathogenicity probabilities) between 0 and 1. As initially defined and referenced in the text, a score of >0.5 indicates that the amino acid substitution is predicted to be deleterious, and a score <0.5 predicts a neutral, or benign, variant. The numerical Rhapsody scores and pathogenicity predictions for all 124 TOPMed ROMK variants and for the 17 variants we selected for functional analysis are outlined in **S1-S3 Tables**. We also uploaded all computed Rhapsody scores that cover the sequences that are included in the ROMK homology model (residues 38-365) to Github: <https://github.com/mgm68/2023_ROMK_LoF/tree/main>. For certain figures and tables (such as **Fig 1** and **S7 Table**), we excluded the numerical scores for clarity. We apologize if the omission of the scores initially caused confusion about the Rhapsody outputs, but we have now included a short sentence in the text (lines 162-164) to address this comment.

4. Does Rhapsody work on AlphaFold models? Are the results for ROMK the same on the AF2 model vs. the homology model?

Since the ROMK homology model has thus far successfully predicted the phenotypic consequences of characterized mutations (e.g., T300R resides in the pore and is indeed channel-dead, and destabilizing mutations reside in a beta-sheet-rich structure), we have been using the homology model, which is based on the crystal structure of Kir2.2, PDB ID: 3SPG, 2.6 Å^1^). A caveat for using the homology model is that it only covers 328/391 amino acids (residues 38-365) in the ROMK structure.

As a result of this limitation, i.e., for variants outside the coverage of the homology model (such as N377K), we utilized the available AF structure to obtain Rhapsody predictions. Thanks to the reviewer’s suggestion, we also performed a comparison of the Rhapsody predictions made with the homology model versus the AF structure for ROMK residues 38-365. Barring the “uncertain” predictions (scores between 0.45 and 0.55), we found that the predictions made with the two structures are in agreement 89% of the time (5252 out of 5805 total predictions). If the uncertain scores are included, then the agreement rate is at 85%. In future studies in which homology models are much more limited, we will publish on these data and a full comparison between homology models and AF in Rhapsody for a diverse range of proteins, including ROMK.

5. Figure S6, ROC curve. It is a little bold to call an AUC of 0.76 "high predictive power". Another challenge is that there are very few datapoints, as seen from the stair-like or rugged nature of the curve. The results section mentions 124 datapoints for ROMK, are these all included here? A ROC curve based on all 127 datapoints is much more convincing than one based on the much smaller subset of variants experimentally tested in this work. Further, the authors should include the ROC curves for other predictors like PolyPhen2 and EVmutation (see also Table S7) to visualise the differences in performance.

We fully acknowledge the reviewer’s concern regarding the AUROC of 0.76 for Rhapsody. This AUROC value was computed based on an ROC analysis of only 18 variants (the control Y314C plus the 17 TOPMed mutations from **Fig 1**), which perhaps explains the rugged nature of the curve as well as the relatively lower AUROC value. We unfortunately cannot perform an ROC analysis with the 124 ROMK variants from **S1 Table**, as we lack yeast growth data for all 124 variants. Thus, true negative/positive metrics are currently unavailable to compute an ROC curve. Regardless, in our previous study to assess Rhapsody accuracy using yeast growth data for 33 variants, the AUROC value was 0.86 for Rhapsody, as opposed to 0.81 and 0.77 for Polyphen-2 and EVmutation, respectively^2^. This is now indicated in the revised text. Furthermore, in the two published papers by the developers of Rhapsody^3,4^, the ROC curves were performed with much larger datasets (12k-20k variants), and the AUROC values were > 0.8. Needless to say, we hope to enlarge our yeast growth datasets for a protein that can be assayed similarly to that outlined in this study, thus allowing for improved ROC calculations based on Rhapsody and yeast growth.

We also note that the AUROC of 0.76 reflects the small dynamic range of our yeast assay for ROMK, as reviewer 1 rightfully pointed out in the first round of reviews. To address this concern, we improved the readout of the yeast growth assays by integrating select mutants into a hyperactive ROMK variant, ROMK-K80M. When these new results are taken into account, the AUROC value improved to 0.81. We thus include this new ROC curve in a significantly expanded version of **S6 Fig** (panel B).

Finally, we computed additional ROC curves using the original set of yeast growth data to compare the accuracy of Rhapsody with other predictive algorithms from **S7 Table**. The ROC curves are shown in the updated **S6 Fig** (panels C-E), with the AUROC values for Polyphen-2, EVmutation, and EVE being 0.83, 0.73, and 0.74, respectively. We did not perform an ROC analysis for SNPs&GO, because this method does not provide numerical scores. Based on the AUROC values, we were heartened to find that Rhapsody (AUROC 0.76) performed well compared to other methods, with the exception of Polyphen-2. We reasoned that Polyphen-2 predictions resulted in a higher AUROC value because: 1) this is a relatively small dataset and 2) Polyphen-2 score distribution is bimodal, with deleterious predictions clustering around 1. With the list of 18 variants being mostly deleterious, this skewed towards a higher AUROC value. Rhapsody, on the other hand, provides a much larger range of “deleterious” scores (i.e., between 0.5 and 1), which we believe aids in the variant selection process for functional analysis. As mentioned above and outlined and cited in the revised text, ROC analyses on Rhapsody were previously performed with much larger datasets, and Rhapsody outperformed both Polyphen-2 and EVmutation.

**Works cited**

1	Hansen, S. B., Tao, X. & MacKinnon, R. Structural basis of PIP2 activation of the classical inward rectifier K+ channel Kir2.2. *Nature* **477**, 495-498 (2011). <https://doi.org:10.1038/nature10370>

2	Ponzoni, L., Nguyen, N. H., Bahar, I. & Brodsky, J. L. Complementary computational and experimental evaluation of missense variants in the ROMK potassium channel. *PLoS Comput Biol* **16**, e1007749 (2020). <https://doi.org:10.1371/journal.pcbi.1007749>

3	Ponzoni, L., Penaherrera, D. A., Oltvai, Z. N. & Bahar, I. Rhapsody: predicting the pathogenicity of human missense variants. *Bioinformatics* **36**, 3084-3092 (2020). <https://doi.org:10.1093/bioinformatics/btaa127>

4	Ponzoni, L. & Bahar, I. Structural dynamics is a determinant of the functional significance of missense variants. *Proc Natl Acad Sci U S A* **115**, 4164-4169 (2018). <https://doi.org:10.1073/pnas.1715896115>
